# Physical Properties and Chemical Characterization of Two Experimental Epoxy Resin Root Canal Sealers

**DOI:** 10.22037/iej.2017.30

**Published:** 2017

**Authors:** Hengameh Ashraf, Farhood Najafi, Soolmaz Heidari, Manijeh Mohammadian, Saeede Zadsirjan

**Affiliations:** a *Department of Endodontics, Dental School, Shahid Beheshti University of Medical Sciences, Tehran, Iran; *; b *Department of Resin and Adhesives, Institute for Color Science and Technology, Tehran, Iran; *; c *Department of Dental Biomaterials, Dental School, Tehran University of Medical Sciences, Tehran, Iran*

**Keywords:** Epoxy Resin, Fourier Transform Infrared, Root Canal Sealer, Scanning Electron Microscopy, X-Ray Energy Dispersive Spectroscopy

## Abstract

**Introduction:**

The aim of this *in vitro* study was to evaluate the setting time, flow, film thickness, solubility, radiopacity and characterization analysis of three epoxy resin based sealers including two experimental sealers and AH-26.

**Methods and Materials:**

Five samples of each material were evaluated for setting time, flow, film thickness, solubility and radiopacity according to ISO 6876 Standard. Characterization of sealers was performed under the scanning electron microscopy (SEM), X-ray energy dispersive spectroscopy, X-ray diffraction (XRD) and Fourier transform infrared (FTIR) spectroscopy. Statistical evaluation was performed using the Kruskal-Wallis test.

**Results:**

In this study, AH-26 showed more radiopacity and flow compared to two other experimental sealers (*P*<0.05). However, both sealers had lower setting time than AH-26 (*P*<0.05). No statistical differences were found regarding film thickness, solubility and radiopacity (*P*>0.05). The characterization analysis exhibited relatively similar microstructure of AH-26 sealer to the experimental root canal sealers.

**Conclusion:**

According to the result of this study, all tested root canal sealers had acceptable properties based on ISO 6876 standard criteria.

## Introduction

Necrosis of the pulp tissue and subsequent microbial infection are the main etiologic factors of apical periodontitis [1]. A disinfected root canal environment can pave the way for periapical healing. Long-term success can be reached with three dimensional filling and coronal restoration, which prevent bacterial leakage [2, 3]. 

Various methods have been recommended for root canal filling [4]. The most frequently used core is semisolid materials such as gutta-percha in combination with root canal sealer or paste [5]. However, gutta-percha alone is not appropriate for ideal root canal filling due to lack of efficient flow and adhesion to canal walls. A satisfactory seal cannot be obtained without the use of a sealer [5, 6]. The different physical and clinical properties of sealers may be examined by laboratory tests: American National Standards Institute/American Dental Association’s (ANSI/ADA) requirements for sealer include radiopacity of at least 3 mm aluminum thickness, less than 3% solubility, more than 20 mm flowability, not more than 50 µm film thickness and setting time that does not exceed 10% of the time specified by manufacturer’s statement [7]. 

Epoxy resin-based sealers were introduced to endodontics by Schroeder [8]. One of these sealers is AH-26 (composed of methenamine and bismuth oxide) [9] which has favorable flow, working time and low solubility and is able to adhere to dentinal walls effectively [9-11].

Assessment of physical properties and characterization analysis of materials are conducted through different ways. Energy-dispersive X-ray spectroscopy (EDX), is an analytical technique used for the elemental analysis or chemical characterization of a sample. It relies on an interaction of some source of X-ray excitation and a sample [12]. 

X-ray Diffraction (XRD) is a technique used for determining the atomic and molecular structure of a crystal, in which the crystalline atoms cause a beam of incident X-rays to diffract into many specific directions. By measuring the angles and intensities of these diffracted beams, a crystallographer can produce a three-dimensional picture of the density of electrons within the crystal. From this electron density, the mean positions of the atoms in the crystal can be determined, as well as their chemical bonds, their disorder, and various other information [13]. 

Fourier transform infrared spectroscopy (FTIR) is a technique used to obtain an infrared spectrum of absorption or emission of a solid, liquid or gas. An FTIR spectrometer simultaneously collects high spectral resolution data over a wide spectral range. This technique shines a beam containing many frequencies of light at once, and measures how much of that beam is absorbed by the sample. Next, the beam is modified to contain a different combination of frequencies, giving a second data point. This process is repeated many times. Afterwards, a computer takes all these data and works backwards to infer what the absorption is at each wavelength [12].

Considering the fact that sealer is an essential material in endodontic treatment, an epoxy resin based sealer with lower price and more appropriate properties is favorable. The purpose of this study was to characterize and evaluate the physical properties of two experimental epoxy resin-based root canal sealers in comparison with AH-26, as the gold standard in this category.

## Materials and Methods

The study was conducted on conventional and experimental root canal sealers: AH-26 (Dentsply, De Trey, Konstanz, Germany), an epoxy resin experimental sealer (ES-A) composed of calcium tungstate, zirconium oxide, aerosil, bismuth oxide, titanium oxide, hexamine and an epoxy resin (Sigma-Aldrich, St Louis, MO, USA) and ES-B with compositions are similar to ES-A except for the presence of imidazoline as a catalyst.

The experimental sealers containing nano-particles were mixed with 37.5% of an epoxy resin. The powder/liquid ratio of ES-A and ES-B sealers were determined by a pilot study. AH-26 (Dentsply, De Trey, Konstanz, Germany) was mixed according to manufacturer’s instructions.

In the present study, setting time, flow, film thickness, solubility and radiopacity of ES-A and ES-B endodontic sealers and AH-26 were measured as outlined in the International Standard ISO 6876 (2012) for dental root canal sealing materials.

***Physical properties analysis***

**Setting time**: The setting time of the sealers was determined according to the ISO 6876 specification and the ASTM C266-0333 standard test [14]. 

The setting time measurements were carried out under controlled temperature and humidity: 37±1^°^C and 95±5% relative humidity. The sealers were mixed and inserted in metallic molds (10 mm in diameter and 2 mm thick). For each sealer five specimens were prepared. After the initial setting time, a Gilmore needle with a weight of 110 g and an active tip of 1.0 mm diameter was used at 5-min intervals to determine the final setting time. The setting times were determined as the time elapsed from the beginning of mixing to the time at which no indentation was detected on the surface of the specimens. Three measurements were performed for each sealer. 

**Flow:** According to ANSI/ADA’s specification [7], after spatulating to obtain a homogenous mixture, 0.5 mL volume of the sealer was placed on a polished glass plate (40×40×5 mm). At 180±5 sec after the commencement of mixing, another plate with a mass of 20±2 g and a load of 100 N was placed carefully and centrally on the top of the plate. Ten min after initiating the mixing, the load was removed and the average of the maximum and minimum diameters of the compressed disc was measured with a digital caliper (Mitutoyo MTI Corporation, Tokyo, Japan). If the difference between both diameters was not more than 1.0 mm, the results were recorded. The film thickness of each sealer was measured three times.

**Film thickness:** Two 5-mm thick glasses were used, and their thickness was confirmed by using a digital caliper. A volume of 0.5 mL of the sealer was placed on the center of one glass plate. The other plate was positioned centrally to the sealer. After 180±10 sec post mixing, a load of 150 N was applied centrally and vertically on top of the plates. Ten min after commencement of mixing, the load was removed, and total thickness of the two plates and the sealer film was measured with a digital caliper. The difference between two measurements showed the film thickness of the materials.

**Solubility:** To determine the solubility, a modified ISO 6876 specification was used. The specimens were molded in accordance to the ISO specification [14] using Teflon ring molds measuring 20 mm in diameter and 1.5 mm high. Five specimens were fabricated for each material. A nylon thread was inserted into the sealers before setting, allowing the sample to be hung and immersed in distilled water throughout the experimental period. The assembly was kept in an incubator at 37^°^C and 9% relative humidity to 3 times of setting time. After setting, the specimens were removed from the molds and any loose material particles were removed from the surface, using a soft brush. Samples were weighed in an analytical balance with 0.0001 g (UMark 210; Bel Engineering, Monza, Italy) precision. The cellophane film was placed on the top of glassware. The samples were suspended by nylon thread and placed inside glassware containing 50 mL of deionized distilled water. Special care was taken to keep the specimens hung in the water, not touching the walls. The containers were stored for 24 h in an incubator (37^°^C and 9% relative humidity). The samples were then removed and gently washed with distilled water, dried with filter paper, placed in oven for 24 h and then weighed again. The experiment was repeated 3 times for each sealer. Solubility was determined by calculating the weight loss (initial mass - final mass), expressed as the percentage of the original mass.

**Radiopacity:** Five cylindrical samples from each sealer were prepared by placing into metallic rings with 10 mm internal diameter and 1 mm thickness. A glass plate was used to ensure that the excess sealer was removed and the top surface was flat. The rings were kept at 37^°^C and 95% relative humidity until cements were completely set. The thickness of each sealer was checked with a digital caliper. The images of the specimens were taken on occlusal films (D-speed, Eastman Kodak Corporation, Rochester, NY, USA), along with aluminum step-wedge (made out 99.5% pure aluminum with thickness varying from 1 to 10 mm). The dental X-ray machine (Planmeca intra, Helsinki, Finland) was used with exposure parameters set at 70 kVp, 10 mA, 3 sec and a focus-film distance of 30 cm. All films were processed in an automatic developing machine (Clarimat, Gendex, USA). Then the radiographs were digitized and analyzed by using Adobe Photoshop CS5 software (Adobe system Incorpoated, San Jose, CA, USA). Each specimen and each step was measured for 10 times. Fifty measurements (ten measurements × five specimens) were calculated to obtain the final density value of each sealer.

***Characterization analysis***

The experimental sealers and AH-26 were characterized using a combination of SEM and EDX, XRD analysis and FTIR. The characteristic analysis was done on both raw and set materials. The set sealers were crushed using a mortar and pestle after setting. The raw and set materials were surface-sputtered with gold and examined using a SEM (Leica Electron Optics, Cambridge instruments, Cambridge, UK) at 8-10 kV and 2-nm resolution. Scanning electron micrographs of the different material microstructural components at different magnifications in back-scatter electron mode were captured and chemical elements were analyzed using the EDX. 

**X-ray diffraction analysis**: Phase analysis of unreacted powders was carried out using XRD. Phase identification was accomplished using a search-match software utilizing ICDD database (International Centre for Diffraction Data, Newtown Square, PA, USA). The diffractometer (Bruker D8 Advance,Bruker Corp., Billerica, MA, USA) was operated in Bragg-Brentano θ-2θ configuration using CuKa radiation at 40 mA and 45 kV and the detector was rotated between 158 and 458 with a step of 0.02^º^2θ and a step time of 0.8 sec.

**Fourier transforms infrared spectroscopic analysis**: FTIR in transmission mode was performed. Set sealers were prepared and powdered using an agate mortar and pestle. For this test 2 to 5 mg of the powdered sealer was mixed with 100 mg potassium bromide and were analyzed in the spectrophotometer (Shimadzu IRAffinity-1; Shimadzu Corp., Kyoto, Japan) using transmitted infrared spectroscopy. 

**Statistical analysis:** Statistical analysis was performed using the Kruskal-Wallis test to evaluate the differences among sealers. For multiple comparisons Mann-Whitney Wilcoxon test was used. Bonferroni correction was applied for significance level. The presence of normal distribution was confirmed in pilot analysis. 

## Results

***Physical properties analysi*** **s**

Mean and standard deviations of the physical tests are shown in Table 1. Experimental endodontic sealers (ES-A, ES-B) presented significantly lower setting time compared to AH-26 (*P*<0.0001). Statistical similarities for film thickness and solubility test were observed among the sealers. For the radiopacity test, AH-26 was found to be the most radiopaque sealer but revealed no statistical difference. Other statistical differences are observed in Table 1.

**Table1:** Means (SD) of physical tests

**Group**	**Setting time (h)**	**Flow (mm)**	**Film thickness (µm)**	**Radiopacity (mm Al)**	**Solubility (%)**
**ES-A**	12.40 (1.577) ^a^	21.9 (1.370)^ a^	24.0 (5.163)^ a^	5.90 (0.20)^a^	0.0053 (0.0006) ^a^
**ES-B**	11.80 (1.475)^a^	23.70 (0.483) ^b^	22.0 (4.216) ^a^	5.99 (0.44) ^a^	0.0051 (0.0013) ^a^
**AH-26**	40.80 (1.032)^b^	25.80 (1.31)^c^	26.0 (6.992)^a^	7.34 (1.62) ^a^	0.0048 (0.0010) ^a^

Different letter in each column indicates statistically significant differences

***Characteristic analysis***

**Figure 1:**
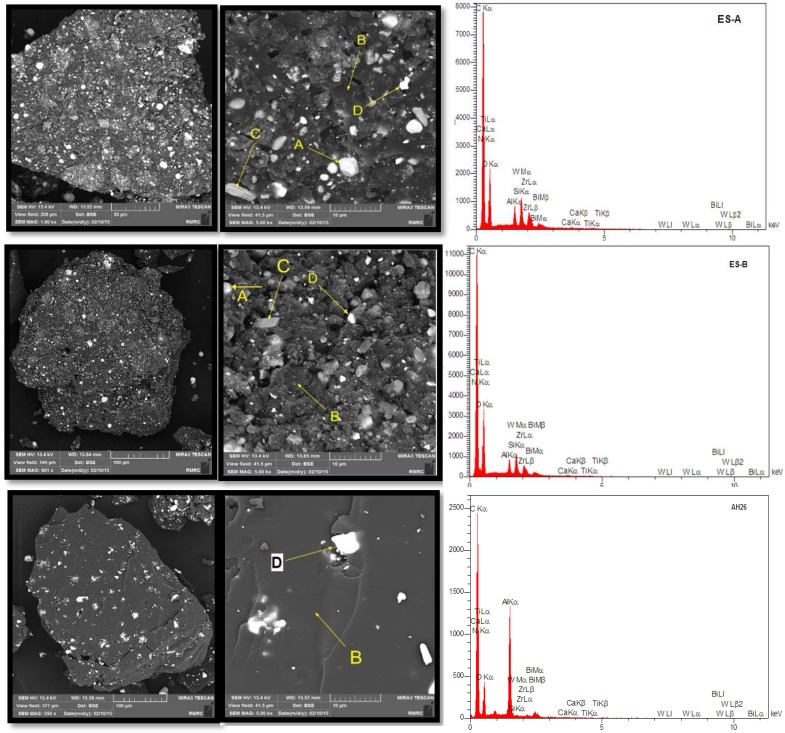
Scanning Electron Micrographs and EDS analysis of set sealers; A) Tungsten, B) Carbon, C) Zirconium and D) Bismuth

**Figure 2:**
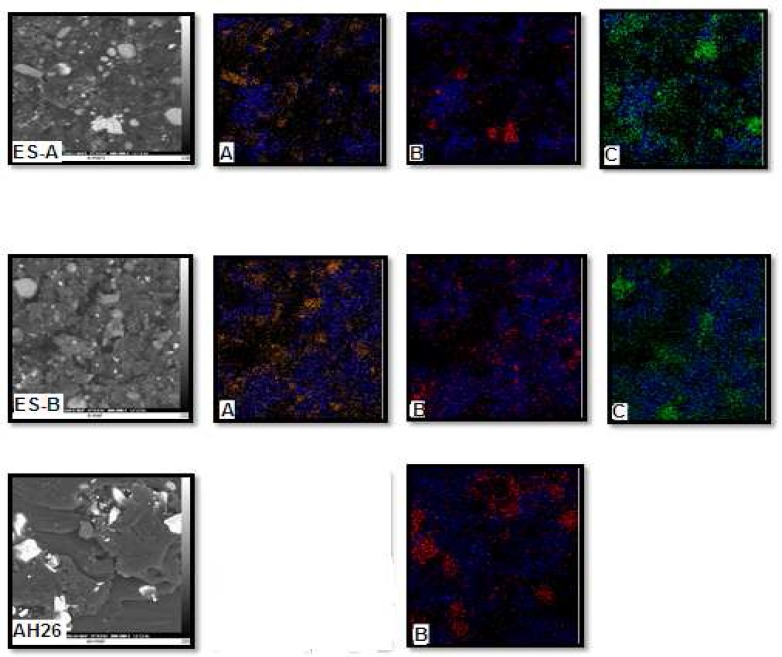
SEM images with ×5000 magnification. *A)* Elements distribution maps of carbon (blue) and zirconium (yellow), *B)* Elements distribution maps of carbon (blue) and bismuth (red), *C)* Elements distribution maps of carbon (blue) and tungsten (yellow

**Scanning electron microscopy and X-ray energy dispersive analysis** ***: ***Quantitative results of elements according to EDX microanalysis are described in Table 2. In Figures 1 and 2, SEM images show the distribution maps of the 2 main elements detected by EDX microanalysis. Experimental root canal sealers contain zirconium oxide, calcium tungstate and bismuth oxide as radiopacifier but the definite radiopacifier in AH-26 is bismuth oxide.

The studied sealers were composed of resin matrix interspersed with shiny particles, 10 µm in diameter, which were rich in calcium and tungsten. Smaller particles present rich in zirconium. The sealer containing the micro-zirconium oxide particles displayed porosity and the cement particles were easily discernible in the resin matrix. AH-26 and experimental sealers had a regular surface and uniformly distributed globular-like particles. 

**X-ray diffraction analysis**: θ-2θ diffraction plots of experimental sealers powder exhibited diffraction peaks for calcium tungstate (ICDD: 41-1431), zirconium oxide (ICDD: 83-0939) and bismuth oxide (ICDD: 41-1449). The AH-26 powder’s diffractogram only exhibited peaks for bismuth oxide (ICDD: 41-1449) (Figure 2). The zirconium oxide (ICDD: 83-0939) and calcium tungstate (ICDD:41-1431) displayed peaks at 28.275^º^, 31.56 ^º^2 Ɵ and 18.608^º^, 28.729^º^, 18.608^ º^2θ.

**Figure 3:**
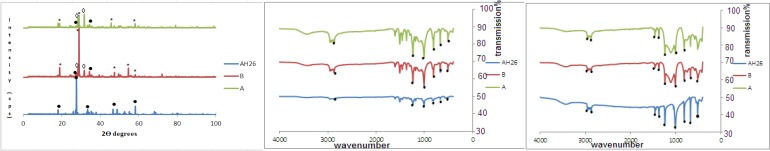
*A)* Powder diffractogram. X-ray diffraction plots of AH-26 and experimental root canal sealers; *B)* Fourier transform infrared spectroscopy plots of test sealers; *C)* powder analysis

The XRD plot of AH-26 exhibited very definite peaks for bismuth oxide (ICDD: 41-1449) at 27.35^ º^, 33.02^ º^ 2 Ɵ. XRD analysis of set materials displayed amorphous structures (Figure 3). 

**Fourier transform infrared spectroscopic analysis:** The FTIR plots of the sealers are shown in **Figure 4**. 

The spectroscopic data show that the infrared spectrum of set materials and powders of three sealers had bands at 510, 670, 815, 1007, 1234 and 2948 cm^-1^ which is related to hexamethylene tetra amine. The infrared spectrum of set sealers had bands related to epoxy resin at 1383, 2923 and 2983 cm^-1^. The bands assigned at 2983 and 2923 cm^-1^, were characterized as C-H stretching, as well as at 1383 cm^-1^, were characterized as CH_2_ deformation. 

## Discussion

The chemical composition of root canal sealers that are used in close contact with periapical tissues is a predictive factor to understand their physical, chemical and biological properties [15]. In the present study, the procedures were performed as outlined in the ISO 6876 guidelines. AH-26 and two experimental endodontic sealers (ES-A and ES-B) are resin-based sealers. In this study, AH-26 showed more radiopacity and flow compared to experimental endodontic sealers (ES-A and ES-B) (*P*<0.05). However ES-A and ES-B had lower setting time than AH-26 (*P*<0.05).

The setting time found in this study was significantly different among ES-A and ES-B and AH-26. In addition, the values presented were within the 10%-variation acceptable by ANSI/ADA [7]. But AH-26 had longer setting time than the manufacturer statements (9-15 h) (Dentsply, DeTrey, Konstanz, Switzerland). This finding is in accordance with Razmi *et al.* [16]. The setting time of ES-A and ES-Band AH-26 sealer were 12.4, 11.8 and 40.8 h, respectively. 

**Table 2:** Elements found in the root canal sealers using energy dispersive X-ray analysis (EDX

	**AH-26**		**ES-B**		**ES-A**	
	**At (%)**	**Wt (%)**	**At (%)**	**Wt (%)**	**At (%)**	**Wt (%)**
**C**	57.39	73.87	65.16	62.69	55.05	65.70
**N**	9.67	6.11	6.28	11.34	11.62	9.49
**O**	26.93	16.75	19.68	24.76	28.97	23.14
**Al**	1.19	2.98	5.90	0.47	0.93	0.61
**Si**	1.19	0.05	0.10	0.40	0.81	0.58
**Ca**	0.16	0.02	0.05	0.06	0.17	0.05
**Ti**	0.25	0.01	0.02	0.04	0.13	0.07
**Zr**	1.62	0.03	0.23	0.17	1.12	0.24
**W**	0.29	0.09	1.18	0.02	0.22	0.02
**Bi**	1.31	0.09	1.38	0.07	0.99	0.09

The variability in setting time is dependent on sealer components, particle size, temperature and relative humidity [17] that in this investigation temperature and humidity were equivalent for three studied sealers. Before the beginning of the study, particle size of experimental sealers were measured with SEM then the similarity in size were confirmed.

The adequate flowability and film thickness are necessary for satisfactory distribution of the sealer into narrow irregularities, lateral canals and the apical foramen [18]. High flow property may result in extruded material over the apical foramen, compromising periodical healing [19, 20]. According to ANSI/ADA’s specification, the sealers should have a diameter not less than 20 mm at flow test and a film thickness not more than 50 µm. High film thickness is an undesirable property due to the possible interference with the proper seating of gutta-percha cones into root canal during filling procedures [21]. In this study, the flow and film thickness of ES-A and ES-B and AH-26 were measured as 21.9 mm and 24 µm, 23.7 mm and 22 µm, and 25.8 mm and 26 µm, respectively. The statistical differences were significant for flowability but not for film thickness. Particle size, film thickness, temperature, rate of insertion, internal diameter of the canal, powder/liquid or paste/paste ratio and shear rate are the factors that influence the flow rate of root canal sealers [22-24]. In this study, all factors could be controlled except shear rate which should be evaluated in next studies.

Solubility is an undesirable property for a root canal sealer because it can cause the release of components that may be biologically incompatible and formation of gaps can affect the hermetic seal of the root canal filling negatively [24]. According to ISO Standards the solubility of root canal sealers should not exceed 3% [7]. In the present study, the solubility of ES-A and ES-B and AH-26 were 0.0053%, 0.0051% and 0.0048%, respectively. This finding confirmed the result of Azadi *et al.* [25]. There were no statistical differences between the tested resin based sealers for solubility. The solubility of AH-26 was low, which was consistent with other studies [10, 22, 26]. However the differences in surface-to-volume values of the specimens as well as other experimental configurations such as molds used and setting time might contribute to the differences in the results [27].

Among other physical and chemical properties, the ideal root canal sealing material should have a certain degree of radiopacity to be clearly visible on radiographs [28, 29]. The radiopacity of root canal sealers should be at least 3 mm aluminum thickness. Radiopacity of AH-26, ES-A, ES-B was 7.34, 5.90, 5.99 mm aluminum, respectively. No significant difference was found between ES-A, ES-B and AH-26 (*P>*0.05). AH-26 exhibited the highest radiopacity of all sealers in this study due to bismuth oxide as a definite radiopacifier as the main component of this sealer. 

In this study, conventional radiography was used to evaluate the radiopacity of root canal sealers. This method was in accordance with previous studies [30, 31]. Total degree of darkening of an exposed film is referred to radiographic density. However, in conventional radiography an unexposed film shows some density owing to the base and added tint as well as development.

***Characterization analysis***

The knowledge of their chemical composition informs the selection of the best material to be used in clinical conditions. In this study, the surface of the specimens showed regularities for each sealer, and a uniform distribution of elements. Surface regularity is important for cellular adhesion and is essential to evaluate biocompatibility [32]. Therefore, better cell adhesion results should be expected when using these root canal sealers. However, other factors, such as chemical composition, may also affect cell adhesion and biocompatibility, and surface regularity should not be analyzed in isolation. EDX microanalysis of the root canal sealers revealed similarities between the elements found in our study and the main compounds described by their manufacturers. All the materials under analysis had elements not described by their manufacturers such as Al. These results might be attributed to contamination during manufacture or to industrial secrets. Resin based sealers may have cytotoxic effects, which may be explained by the fact that its main component is epoxy resin, and that it releases amines, or formaldehyde [33]. The high amounts of zirconium and tungsten may explain part of this cytotoxic mechanism.

The sealer microstructure and chemical composition was determined by several techniques. The XRD provides detailed information on the crystallographic structure and can be used to identify the phase composition of solid [34]. XRD analysis only detects regular structures (crystalline) in the composition of the test materials or precipitates while amorphous structures cannot be identified [35]. 

On the other hand, the FTIR is an unspecific technique that only identifies functional chemical groups of the chemical components and each functional group absorbs a specific wavelength of radiation in the infrared region. Consequently, the graph of radiation intensity versus frequency (spectrogram) allows the characterization of the functional groups of standard or unknown material [36]. In this study, similarity in peaks between experimental sealers and AH-26 in spectroscopic data showed that the experimental sealers structure was close to AH-26.

## Conclusion

According to the result of this study, all root canal sealers showed acceptable properties based on ISO 6876 standards.
